# The LexA regulated genes of the *Clostridium difficile*

**DOI:** 10.1186/1471-2180-14-88

**Published:** 2014-04-08

**Authors:** Beata M Walter, Maja Rupnik, Vesna Hodnik, Gregor Anderluh, Bruno Dupuy, Nejc Paulič, Darja Žgur-Bertok, Matej Butala

**Affiliations:** 1 Institute of Public Health Maribor, Centre for Microbiology, Maribor, Slovenia; 2 Faculty of Medicine, University of Maribor, Maribor, Slovenia; 3 Centre of Excellence for Integrated Approaches in Chemistry and Biology of Proteins, Ljubljana, Slovenia; 4 Biotechnical Faculty, University of Ljubljana, Department of Biology, Ljubljana, Slovenia; 5 National Institute of Chemistry, Ljubljana, Slovenia; 6 Laboratoire Pathogenèse des Bactéries Anaérobies, Département de Microbiologie, Institut Pasteur, Paris, France

**Keywords:** *Clostridium difficile*, Antibiotic resistance, Toxin regulation, SOS system, Surface plasmon resonance, LexA repressor

## Abstract

**Background:**

The SOS response including two main proteins LexA and RecA, maintains the integrity of bacterial genomes after DNA damage due to metabolic or environmental assaults. Additionally, derepression of LexA-regulated genes can result in mutations, genetic exchange and expression of virulence factors. Here we describe the first comprehensive description of the *in silico* LexA regulon in *Clostridium difficile*, an important human pathogen.

**Results:**

We grouped thirty *C. difficile* strains from different ribotypes and toxinotypes into three clusters according to *lexA* gene/protein variability. We applied *in silico* analysis coupled to surface plasmon resonance spectroscopy (SPR) and determined 16 LexA binding sites in *C. difficile*. Our data indicate that strains within the cluster, as defined by LexA variability, harbour several specific LexA regulon genes. In addition to core SOS genes: *lexA*, *recA*, *ruvCA* and *uvrBA*, we identified a LexA binding site on the pathogenicity locus (PaLoc) and in the putative promoter region of several genes involved in housekeeping, sporulation and antibiotic resistance.

**Conclusions:**

Results presented here suggest that in *C. difficile* LexA is not merely a regulator of the DNA damage response genes but also controls the expression of dozen genes involved in various other biological functions. Our *in vitro* results indicate that in *C. difficile* inactivation of LexA repressor depends on repressor`s dissociation from the operators. We report that the repressor`s dissociation rates from operators differentiate, thus the determined LexA-DNA dissociation constants imply on the timing of SOS gene expression in *C. difficile*.

## Background

Organisms have evolved gene regulatory systems to maintain their genetic integrity. The SOS regulatory network is a paradigm for bacterial response to DNA damage which is controlled by a global transcriptional repressor LexA and an inducer, the recombinase protein RecA. During normal bacterial growth, LexA binds to DNA recognition sequences (operator) positioned near or overlapping the promoter elements of the SOS genes and occludes RNA polymerase, preventing SOS gene transcription. Upon DNA damage, RecA polymerizes on single-stranded DNA (ssDNA) formed at sites of DNA damage, becomes activated (RecA*) and facilitates self-cleavage of LexA resulting in coordinated expression of SOS genes [1].

The SOS system was found in almost all eubacterial groups [2]. It was suggested that the LexA operator spread from Gram positive bacteria into Gram negative bacteria, which indicates on the evolutionary origin of the LexA protein [3]. In *Escherichia coli*, the consensus operator sequence (SOS box) has been identified as 5′-CTGTN_8_ACAG-3′ [4] and in the spore former *Bacillus subtilis* 5′-GAACN_4_GTTC-3′ [5]. The SOS response comprises a variety of physiological processes, not solely involved in the upkeep of the bacterial genome. LexA represses synthesis of toxins [6,7] and antibiotic resistance determinants [8], controls integron cassette recombination [9] and lateral transfer of virulence factor genes [10], as well as drug resistance genes [11].

Genes under the control of LexA differ significantly among species. *B. subtilis* LexA controls a regulon of over 60 genes [12] with only eight of these genes having orthologs in *E. coli*. Those genes play roles in SOS regulation and excision, recombinational and error-prone DNA repair [5].

*C. difficile* is a human pathogen causing a spectrum of intestinal diseases ranging from mild diarrhoea associated with antibiotic treatment to, in more severe cases, pseudomembraneous colitis [13]. Despite extensive research focused on the bacterium, knowledge regarding its SOS system is scarce [14]. Among other clostridia species, binding sites for LexA were identified in *C. acetobutylicum* and *C. perfringens* and resemble *Bacillus* LexA operator sequences [15,16]. As a suitable target site for LexA is sufficient for binding *in vivo*[4], we used a robust *in silico* approach [17] and predicted the LexA-regulated genes of several *C. difficile* strains. In addition, surface plasmon resonance (SPR) was used to confirm the interactions of LexA with regions defined in *in silico* experiments.

## Results and discussion

### Variability of the *lexA* gene in *C. difficile*

**Figure 1:**
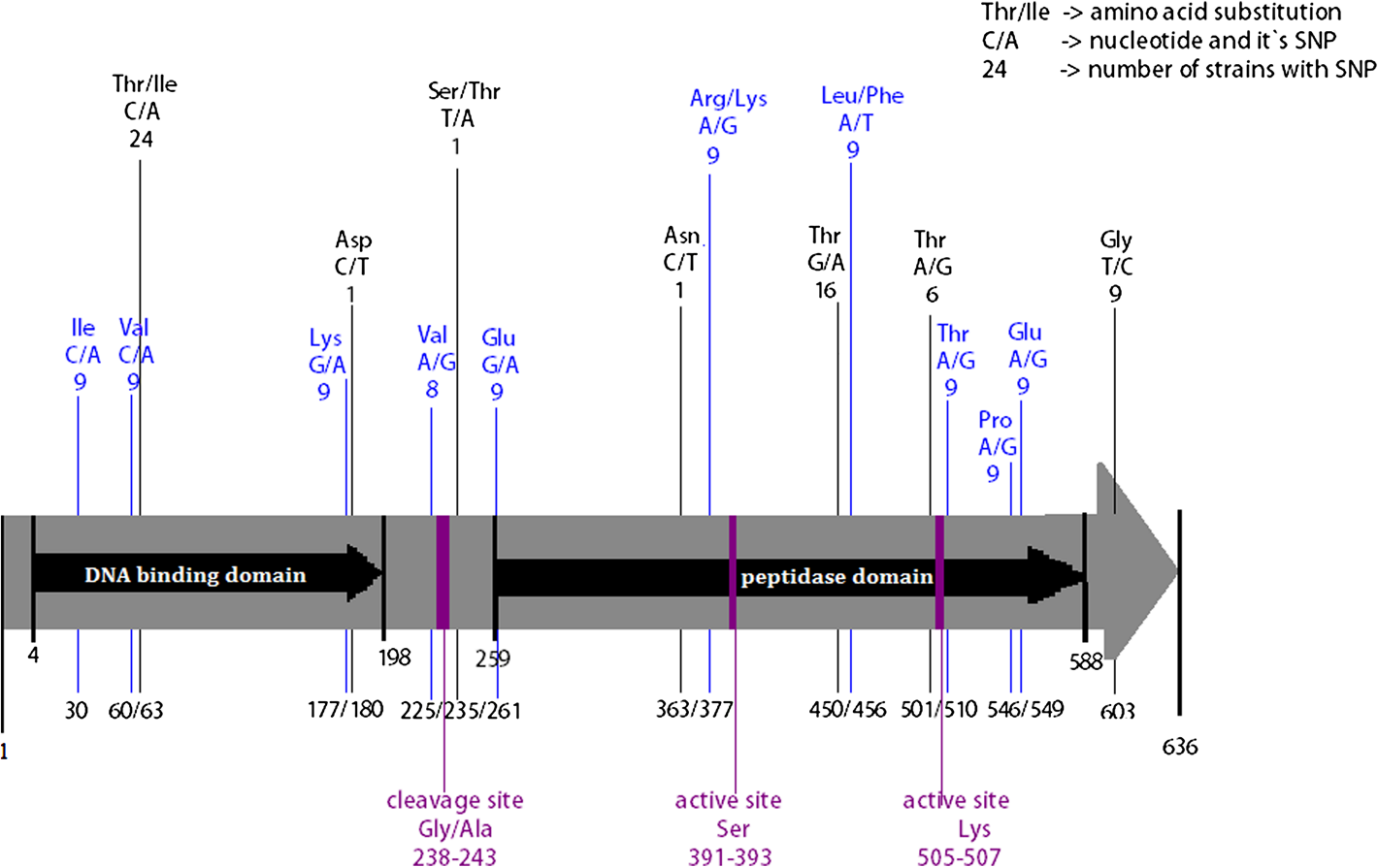
**Variability of *lexA *gene *in Clostridium difficile*.** Representation of the *C. difficile* 630 strain *lexA* nucleotide sequence in comparison to repressor sequences of 62 other strains. Grey arrow denotes the nucleotide sequence of the CD630 *lexA* gene. Black arrows mark the position of domains in LexA. The number of strains with specific SNP and the corresponding nucleotide/aminoacid change is marked above the arrow. The ordinal number of nucleotides in *lexA* is presented below the arrow. The SNPs marked in blue encompass strains from cluster III, composed mainly of strains belonging to the toxinotype V. The position of the cleavage site and the catalytic residues is marked in purple.

**Figure 2:**
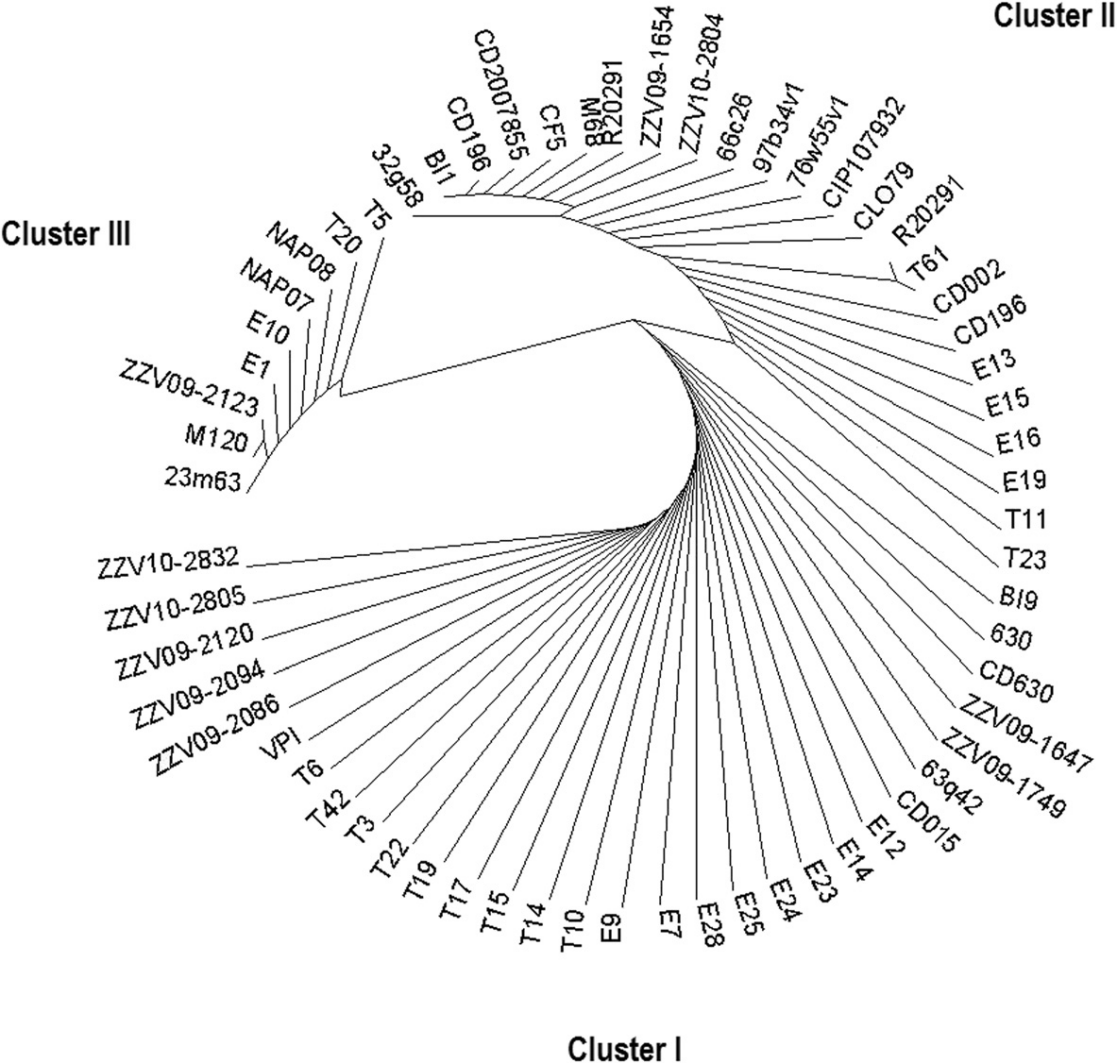
**Dendrogram of the aminoacid sequence allignments of LexA derived from *lexA *genes of *C. difficile *strains.** PCR ribotypes and toxinotypes of the strains can be found in Additional file 1.

*C. difficile* has been described as a bacterium with highly mosaic genetic composition and multiple attempts have been made to distinguish between various strains and to correlate them with virulence [18]. We first analysed the variability of the repressor LexA encoding gene sequence among various *C. difficile* ribotypes (groups characterized by differences in intergenic regions of RNA operon and used worldwide for *C. difficile* typing) and toxinotypes (characterized by differences in toxin A and B coding region inside the pathogenicity locus called PaLoc) (Additional file 1: Table S1) [19]. Analysis revealed 17 single nucleotide polymorphisms (SNPs) in the *lexA* gene of 63*C. difficile* sequences among which four SNPs resulted in missense mutations but none of the mutations modified amino acids in the cleavage or active sites of LexA (Figure 1). Our analysis grouped the investigated strains into three clusters according to the *C. difficile* LexA (Figure 2). Cluster I encompassed 3 non-toxinogenic strains and strains of toxinotype 0; Cluster II encompassed strains of toxinotypes III, VIII, IX, and X and finally, Cluster III with the highest number of SNPs, was mostly composed of toxinotype V strains. Ribotypes for the above stated toxinotypes can be found in the Additional file 1: Table S1. Previous results showed that strains belonging to the epidemic ribotype 027 form a genome wide clade [20,21], typically characterised as the toxinotype III (North American pulsed field gel electrophoresis type 1 - NAP1, REA group BI). Interestingly, ribotypes 016, 019, 036, 075, 111, 122, 153, 156, 176, 208 and 273 are closely related to ribotype 027 by comparative genomics [20,21], and those ribotypes were found to encompass the *lexA* cluster II. Comparative phylogenomics along with MLST (multilocus sequence typing) and whole genome sequecing has shown that ribotype 078 lineage is different than other *C. difficile* lineages [22]. Moreover PCR ribotype 078 forms a phylogenetically coherent group with ribotypes 033, 045, 066, 078, 126 and 127 [23] – which encompasses *lexA* cluster III. Genetically distinct strains that belong to ribotypes 078 (V) and 126 (V) clustered together showing the highest number of SNPs in the *lexA* gene. The phylogenetic tree based on LexA variability reflects similarities to genetic lineages based on ribotype patterns and comparative genomics analysis.

### *In silico* screening for the LexA-regulated genes in *C. difficile*

To obtain insight into the LexA regulon genes, we performed *in silico* genome-wide prediction of LexA binding sites within promoter regions of *C. difficile*. Using the xFiToM software [24], we screened genomes of thirty *C. difficile* strains (Additional file 1: Table S1) for the *C. acetobutylicum* and *C. perfringens* consensus operator sequence of LexA [15,16], allowing for two mismatches in one of the two half sites positioned within 350 bp upstream to 35 bp downstream of a protein coding sequence. Among the thirty genomes, the search yielded at least one putative operator sequence upstream of more than 30 genes involved in a variety of biological processes e.g. DNA repair, transport, virulence and antibiotic resistance (Table 1).

**Table 1:** ***In silico *** **predicted LexA binding sites in *C. difficile *** **ribotypes**

					**Various toxinotypes**				**Toxinotype V**		**Toxinotype 0/nontoxinogenic**						
					**O33**	**O27**	**O75**	**O17**	**O78**	**126**	**OO9**	**OO1**	**O12**	**OO5**	**O87**	**O14**	**O53**
**Gene accession number**	***GENE***	**Product**	***LexA BOX***	**Distance**	**1 strain**	**8 strains**	**2 strains**	**1 strain**	**3 strains**	**2 strains**	**1 strain**	**3 strains**	**3 strains**	**3 strains**	**1 strain**	**1 strain**	**1 strain**
CDR20291_1854	***lexA***	Transcriptional regulator. LexA repressor	GAAC....GTTT	−**51/**-**91**	1	8	2	1	3	2	1	3	3	3	1	1	1
CDR20291_1169	***recA***	Protein RecA (Recombinase A)	GAAC....GTTT	−**39/**-**41**	1	8	2	1	3	2	1	3	3	3	1	1	1
CDR20291_2696	***ruvC***	Crossover junction endodeoxyribonuclease	GAAC....GTTT	−**65**	1	8	2	1	3	2	1	3	3	3	1	1	1
CDR20291_3234	***uvrB***	Excinuclease ABC subunit B	GAAC....GTTC	−**30**	1	8	2	1	3	2	1	3	3	3	1	1	1
CDR20291_0487	***rusA***	Putative RusA-like endodeoxyribonuclease	GAAC....GTTT	−**122**	1	4	1	1	3	2	NO	NO	1	NO	NO	1	NO
CDR20291_2024	***trxB***	Thioredoxin reductase	GAAC....GTTT	−**216**	NO	NO	NO	NO	NO	NO	1	NO	NO	NO	NO	NO	NO
63q42v1_580022	***rps3***	Putative 30S ribosomal protein S3	GAAC....GTTA	−**284**	NG	NG	1	NG	NG	NG	NG	1	NG	NG	NG	NO	NO
CDR20291_3107	***sspB***	Small. acid-soluble spore protein beta	GAAC....GTTC	34	1	8	2	1	3	2	1	3	3	3	1	1	1
CDR20291_0784	***oppC***	ABC-type transport system. oligopeptide	GAAC…GTTT	−**285/-286**	1	8	2	1	3	2	1	3	3	3	1	1	1
CDR20291_3532	***soj***	Small walker A ATPase, chromosome replication	GAAC....GTTT	−**226**	NO	8	2	1	NO	NO	1	3	3	3	NO	1	1
CDR20291_2297		Putative multidrug efflux pump	GAAC…TTTT	−**138**	1	8	2	1	3	2	1	3	3	3	1	1	1
63q42v1_310170		ABC-type multidrug-family	GAAC....CTTT	−**154**	1	8	2	1	3	2	1	3	3	3	1	1	1
CDR20291_3125	***vanR***	Regulatory protein vanR	GAAC....ATTT	−**222**	NO	8	2	NO	NO	NO	NO	NO	NO	NO	NO	NO	NO
CDR20291_0083	***rplR***	50S ribosomal protein L18	GAAC....GTTT	−**261/-262**	1	8	2	1	3	2	1	3	3	3	1	1	1
CDR20291_0060	***rpoB***	DNA-directed RNA polymerase subunit β	GAAC…GTTT	−**42**/-**43**	1	8	2	1	3	2	1	3	3	3	1	1	1
CDR20291_1619		Putative transcriptional regulator	GAAC…GTTT	**30**/**31**	1	8	2	1	3	2	1	3	3	3	1	1	1
63q42v1_570034		Helix-turn-helix domain protein	GAAC…CTTT	−**97**	NG	3	NG	1	NG	NG	NG	1	NG	1	NG	NG	NG
CDR20291_0882	***potC***	ABC-type transport system.	GAAC…GTTC	−**207**	1	8	2	1	3	2	1	3	3	3	1	1	1
CDR20291_0584	***tcdA***	Toxin A	GAAC....GTTT	−525	NG	8	2	NG	3	2	NG	3	3	3	1	1	1
CDR20291_3466		Putative cell wall hydrolase	GAAC…GTTT	−**68**	NO	8	NG	NO	NO	NO	NO	NO	NO	NO	NO	NO	NO
CDR20291_2689		Putative membrane protein	GAAC....GTTT	−**111**	NO	7	2	1	NO	NO	1	3	3	3	1	1	1
CDR20291_1611	***moaB***	Molybdenum cofactor biosynthesis	GAAC…GTTT	−**6**	NO	8	2	NO	NO	NO	1	3	NO	3	1	1	NO
CDR20291_2775	***celG***	Cellobiose-phosphate degrading protein	GAAC....GTTT	−314	NO	8	2	NO	NO	NO	NO	NO	NO	NO	NO	NO	NO
CDR20291_3372	***phnH***	Phosphonate metabolism protein	GAAC....CTTT	−**34**	NG	8	2	1	NG	NG	1	3	3	3	1	1	1
CDR20291_1600	***thiC***	Thiamine biosynthesis protein ThiC	GAAC....ATTT	−**175**	1	NO	NO	NO	3	2	NO	NO	NO	NO	NO	NO	NO
CDR20291_1940		N-carbamoyl-L-amino acid hydrolase	GAAC....GTTT	−**147**	NO	NO	NO	NO	NO	NO	NO	3	3	NO	NO	NO	1
CDR20291_2056		Endonuclease/exonuclease/phosphatase	GAAC....GTTT	−466	1	8	2	1	3	2	1	3	3	3	1	1	1
NAP07v1_640016		Two-component sensor histidine kinase	GAAC....GTTT	−**217**	NO	8	NO	NO	NO	NO	NO	NO	NO	NO	NO	NO	NO
CDR20291_0331	***cbiQ***	Cobalt transport protein	GAAC....GTTT	−**122**	1	8	2	1	3	2	1	3	3	3	1	1	1
CDR20291_2597		Putative oxidoreductase	GAAC....CTTC	**2**	1	8	2	1	3	2	1	3	3	3	1	1	1
NAP07v1_470051	***aroF***	P-2-dehydro-3-deoxyheptonate aldolase	GAAC....CTTT	−**225**	1	NO	NO	NO	3	2	NO	NO	NO	NO	NO	NO	NO
97b34v1_600001		Transposase	GAAC....GTTT	−**217**	NO	8	NO	NO	NO	NO	NO	NO	NO	NO	NO	NO	NO
CDE15v2_1270013		Putative cI repressor	GAAC....GTTC	−**67**	NG	NG	NG	NG	NG	NG	NG	NG	NG	NO	1	NO	NG
63q42v1_370450		Extrachromosomal origin protein	GAAC…GTTT	10	NG	NG	NG	NG	NG	NG	1	3	3	3	1	1	1
CDR20291_1803	***vexP***	ABC transporter. ATP-binding/permease	GTTC....TTTT	−**85**	NO	8	2	1	NO	NO	NO	1	2	NO	NO	NO	1
97b34v1_250108		ABC-type transport system. sugar-family	GAAC…GTTC	−**267**	NG	8	2	NG	NG	NG	NG	NG	NG	NG	NG	NG	NG

Sequences of putative LexA operators and their positions (according to the start of the gene coding region). Numbers denote strains with the operator identified. NO marks the gene that was identified in the strain but a target LexA site was not found in its promoter region, NG marks that gene was not found in the genome of the strain.

**Figure 3:**
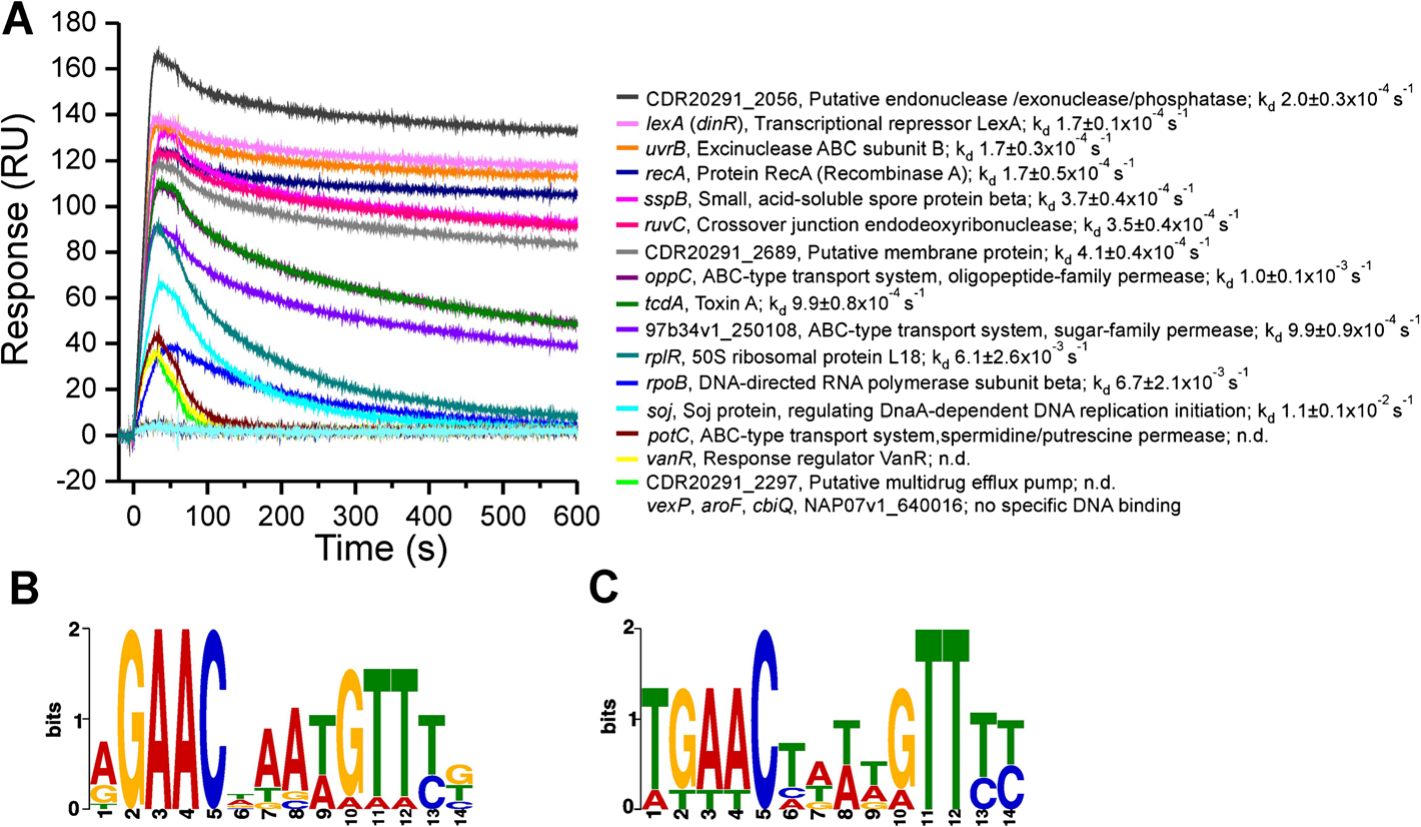
***C. difficile *LexA regulon genes. (A)** SPR sensorgrams of the binding of *C. difficile* LexA with *in silico* predicted target DNA sites. Selection of LexA target genes determined by *in silico* and analysed by SPR. LexA (20 nM) was injected for 60 s across the chip-immobilized DNA fragments containing either of the putative operators and dissociation was followed for 540 s. The representative sensorgrams are shown and the dissociation constants presented as average values of triplicate experiments presented with standard deviation. By n.d. we mark if dissociation rate constant was not determined and the response units are marked by RU. With the MEME tool determined motifs for the target DNA sites found in promoter regions of the genes higher affinity CDR20291_2056, *lexA*, *uvrB*, *recA*, *sspB*, *ruvC*, CDR20291_2689, *oppC*, *tcdA*, 97b34v1_250108, showing high affinity for LexA **(B)** or of the genes *rplR*, *rpoB*, *soj*, *potC*, *vanR*, CDR20291_2297 to which LexA does not bind stably **(C)**.

Subsequently, we purified *C. difficile* LexA and RecA proteins with an N-terminal hexa-histidine tag (Additional file 2: Figure S1) as described for *E. coli* orthologs [25]. SPR analysis was performed to validate the *in silico* data and determine the LexA-operator interactions *in vitro* in real time. Most of the interaction sites were found in putative promoter regions of “common” putative SOS genes for the majority of the genomes tested and of putative LexA regulon genes encoding unusual SOS proteins. Out of 20 DNA fragments tested, the repressor interacted with 16 targets (Figure 3A, Additional file 3: Table S2). We determined interaction with operators in promoter regions of the core SOS response genes: *recA*, *lexA*, the genes of the *uvrBA* operon encoding for components of the UvrABC endonuclease catalyzing nucleotide excision repair and the *ruvCA* operon genes, encoding the nuclease that resolves Holliday junction intermediates in genetic recombination. In addition, LexA interacts with putative promoter regions of genes involved in sporulation (*sspB*), regulation of DnaA-dependent initiation of DNA replication (*soj*), several ABC transporters (*potC*, *oppC*, MicroScope:CDR20291_2297) and for homologue of a two-component system regulator of the vancomycin resistance cluster (*vanR*). The LexA repressor was also found to interact within PaLoc with operator identified 525 base pairs upstream of the toxin A gene (*tcdA*). While the regulation of toxin production in *C. difficile* is controlled in response to several environmental signals mediated by pleiotropic regulators (CcpA, CodY, SigD and SigH [26]), the possible regulation through the SOS system sheds new light on this issue. Furthermore, the subinhibitory concentration of SOS-inducing antibiotic ciprofloxacin was recently shown to increase the Toxin A gene expression in *C. difficile*[27]. Our SPR analysis revealed that also housekeeping genes required for ribosome function (*rplR*) and β subunit RNA polymerase (*rpoB*) belong to the LexA regulon, a feature of the SOS network not yet observed in bacteria. Thus, blockage of LexA self-cleavage could impede pivotal functions in *C. difficile* and this might provide a new approach to treat *C. difficile* infections. Moreover, although putative SOS genes are present in most of the analysed genomes, several of these genes encoding for putative cell wall hydrolase, transposase and for two component sensor histidine kinase seem to be regulated by LexA only in the 027 ribotype strains (Table 1). The *in silico* analysis showed operators in front of several genes upregulated exclusively in ribotype 075 and 027 (*celG*, *vanR*, ABC-type transport system). Furthermore, among the analysed genomes, exclusively in the closely related ribotypes 078, 126 and 033, the LexA target site was not found in front of the *soj* (regulation of DNA replication) and the *phnH* (phosphonate metabolism protein). Thus the mode of SOS regulation might be related to phylogenetic lineages.

### Cross-reaction of SOS system components in *E. coli* and *C. difficile*

**Figure 4:**
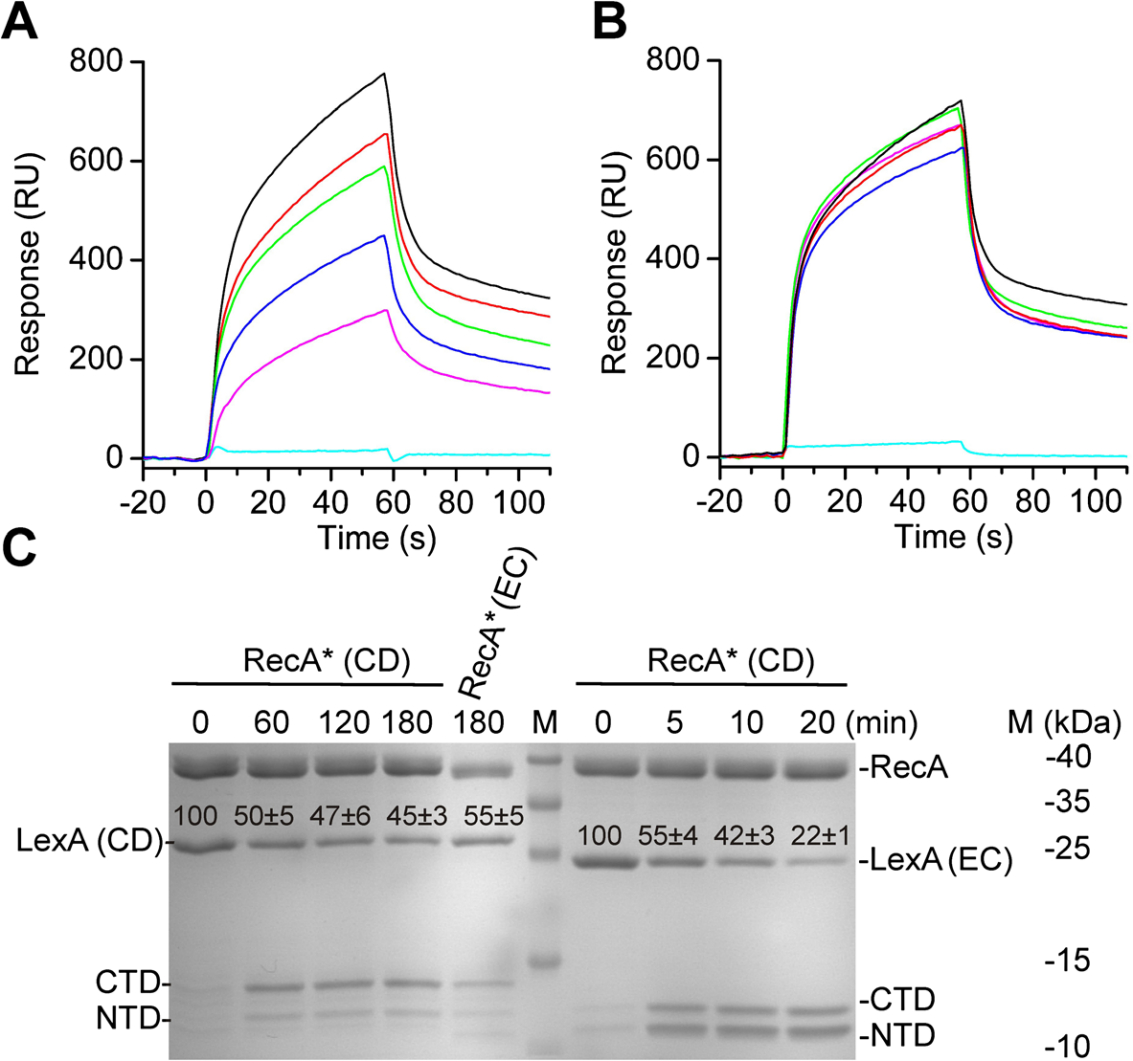
Specific DNA precludes *C. difficile *RecA*-**LexA interaction.** Interaction of *C. difficile* LexA repressor (2.6 μM) incubated with specific, 22-bp *recA* operator **(A)** or with non-specific DNA fragment, *recA* operator with modified six nucleotides **(B)**, with the chip-immobilized *C. difficile* RecA* (~2000 response units). The used DNA interacting with repressor was in 1.4 μM (black line), 2.7 μM (red line), 4.0 μM (green line), 5.4 μM (blue line), 8.1 μM (pink line) concentration. The cyan line presents sensorgram of the free DNA at 8.1 μM concentration interacting with the RecA*. **(C) ***In vitro* repressor cleavage pattern exhibits that purified *E. coli* and *C. difficile* key SOS players can cross-react. *C. difficile* proteins are marked as RecA* (CD), LexA (CD) and *E. coli* proteins as RecA* (EC) and LexA (EC), respectively. Time course (min) of either *C. difficile* or *E. coli* RecA*-induced inactivation of LexA (CD) or LexA (EC) repressor. Quantification of LexA is presented on the gel above the respective band as the ratio (%) of the protein density value of the initial sample (0 min) relative to the density value obtained from the proteins after indicated time points after addition of RecA*, shown with standard deviation.

Induction of SOS gene expression is synchronized and the level, timing and duration of expression of the individual LexA regulon genes differs significantly (1). *In E. coli*, LexA bound to target DNA cannot interact with RecA* and only unbound repressor is proteolytically inactivated [25]. Thus the rate of LexA dissociation from operators controls the precise timing of SOS gene expression following induction. Consequently genes with lower affinity LexA target sites are expressed prior to genes with high affinity operators [1,5]. To follow up on these results, we used SPR to study interaction of the chip-immobilized *C. difficile* RecA* with LexA interacting with either specific or non-specific DNA. We showed that as in *E. coli*, the *C. difficile* LexA repressor interaction with RecA* is prevented by binding to specific DNA targets (Figure 4). In addition, we showed that the key SOS players of *E. coli* and *C. difficile* can cross-react *in vitro* (Figure 4). Hence, our data indicated that the mode of regulation of the *C. difficile* SOS response resembles the one described for *E. coli*. Nevertheless, in contrast to the *E. coli* SOS system, we observed among the investigated *C. difficile* genes, a slowest LexA dissociation from operators of the core SOS genes, *recA*, *lexA* and *uvrB* (Figure 3A and B, Table 2), implying that these are the last genes upregulated upon SOS induction. For instance, LexA dissociation from the *E. coli recA* operator is more than 20-times faster than from *C. difficile* with regard to the dissociation constants of 4.8 ± 2.1 × 10^−3^ s^−1^ (21) and 1.7 ± 0.5 × 10^−4^ s^−1^, respectively.

**Table 2:** Target DNA sequences of the putative SOS genes of the R20291 strain used for the SPR analysis

**GENE**	**Function**	**Product**	**Putative LexA operator (R20291 strain) (5`- -3`)**	**Distance from CDS**
***lexA***	SOS response	Transcriptional regulator. LexA repressor	AG**GAAC**AAAT**GTTT**GC	−51/-91
***recA***	SOS response/DNA repair	Protein RecA (Recombinase A)	GA**GAAC**AAAT**GTTT**GT	−39/-41
***ruvC***	DNA repair	Crossover junction endodeoxyribonuclease	TA**GAAC**ATAA**GTTT**TT	−65
***uvrB***	DNA repair	Excinuclease ABC subunit B	AG**GAAC**TAAT**GTTC**GA	−30
***sspB***	Spores	Small. acid-soluble spore protein beta	CA**GAAC**AGTA**GTTC**CA	34
***oppC***	Spores/ABC transporter	ABC-type transport system. oligopeptide-family	TA**GAAC**ATAA**AAAT**TT	−285/-286
***soj***	Regulation of DNA replication	protein Soj	TT**GAAC**TTTA**GTTT**CT	−226
**CDR20291**_**2297**	Antibiotics	Putative multidrug efflux pump	AA**GAAC**ATCT**GAAA**AG	−138
***vanR***	Antibiotics	Response regulator VanR	CA**GAAC**TATT**ATTT**TA	−222
***rplR***	DNA/RNA	50S ribosomal protein L18	AT**GAAC**TTAG**GTTT**CT	−261/-262
***rpoB***	DNA/RNA	DNA-directed RNA polymerase subunit beta	AT**GAAC**TATT**GTTT**TA	−42/-43
***potC***	Biofilm	ABC-type transport system. spermidine/putrescine	TG**GAAC**TTTG**GTTC**AG	−207
***tcdA***	Toxicity	Toxin A	GT**GAAC**CAAT**GTTT**GA	−525
**CDR20291**_**2689**	Cell wall/membrane	Putative membrane protein	TG**GAAC**TTTA**GTTC**TA	−111
**CDR20291**_**2056**	Signalling	Putative endonuclease/exonuclease/phosphatase	AA**AAAC**ACCC**GTTC**TGC**AAAC**ATTC**GTTC**TG	−466
**NAP07v1**_**640016**	Signalling/Chemotaxis	Two-component sensor histidine kinase	GA**GAAC**CTGT**GTTT**TT	−217
***cbiQ***	Transport	Cobalt transport protein	AT**GAAC**CATG**GTTT**AG	−122
***aroF***	Transport	Phospho-2-dehydro-3-deoxyheptonate aldolase	AT**GAAC**TATT**CTTT**CT	−225
***vexP***	ABC transporter	ABC transporter. ATP-binding/permease protein	AA**GTTC**AAAT**TTTT**GA	−85
**97b34v1**_**250108**	ABC transporter	ABC-type transport system sugar-family	AA**GAAC**TAAA**GTTC**CT	−267

We propose that in *C. difficile*, strong repression of core SOS genes affects the magnitude of the system`s induction. Thus, the low association and non-stable LexA binding to putative regulatory regions of genes encoding the RNA polymerase β subunit (*rpoB*), 50S ribosomal protein (*rplR*), spermidine/putrescine permease (*potC*), vancomycin response regulator (*vanR*) and putative multidrug-efflux-pump [MicroScope: CDR20291_2297], indicates that LexA contributes to fine-tuning of expression of these genes independently of substantial *recA* induction (Figure 3).

The paradigm of the SOS system is that DNA repair genes are rapidly induced in the SOS response to deal with DNA lesions [1,2,28]. However, comparison of induction of LexA regulon genes in *B. subtilis* and *E. coli* in response to double-strand breaks reveals diversity [29]. After DNA damage, the velocity of assembly of RecA* is similar but in contrast to *E. coli*, a limited set of LexA-regulated genes are induced early in the response in *B. subtilis*. Our *in vitro* results suggest that also in *C. difficile*, induction of the LexA-regulated DNA repair genes might be induced later in the SOS response as the core SOS gene promoter regions harbour high affinity LexA targets. According to the differences in LexA-operator affinities we predict that upon DNA damage, various biological processes will be derepressed without induction of the SOS DNA repair.

## Conclusions

We have generated maps of LexA target sites within the genomes of *C. difficile* strains. We predict that SOS functions in *C. difficile* are not solely involved in the DNA repair but are probably linked to other biological functions (virulence factors, sporulation,…). As *C. difficile* infection is a growing problem in healthcare facilities and community patients, further characterisation of the LexA-regulon could provide key insights into pathogenesis. Our data suggest that molecules targeting key SOS proteins could block several houskeeping functions and could provide next generation of *C. difficile* antibiotics. Furthermore, the defined differences in *lexA* gene group *C. difficile* strains into three clusters which correlated well with phylogentic lineages suggested by comparative genomic approaches.

## Materials and Methods

### Source

The *C. difficile* genomes were obtained from an opened access NCBI database [30] and an undisclosed access to MicroScope platform [31]. The strains used for amplification with PCR and sequencing belong to the strain collection of the Institute of Public Health Maribor. The list of strains used for analysis of the LexA variability and regulon is presented in the Additional file 1: Table S1.

### *Variability* of *lexA* gene

Variability of *lexA* in *C. difficile* was compared by analysis of alignment and phylogenetic trees of nucleotides and amino acid sequences performed with Vector NTI (Invitrogen) and with the interactive viewer for phylogenetic trees: Dendroscope [32]. Sixty three sequences were analysed in total (NCBI – 9 strains, MicroScope – 44 strains, PCR product of in-house strains – 10). Strains CD196, R20291 and 630 were obtained from both databases. List of strains used for *lexA* gene variability can be found in Additional file 1: Table S1.

### *In silico* determination of the *C. difficile* SOS regulon

The search for LexA binding sites was performed for 30 genomes (Additional file 1: Table S1). The number of strains covering ribotypes was as follows: ribotype 027 – eight strains; ribotypes: 078, 001, 005 and 012 - three strains from each; ribotypes 075 and 126 two strains from each and one genome from each ribotypes 017, 087, 014, 053. The analysis was performed with xFiToM software [24]. The searched motifs, based on *C. acetobutylicum* and *C. perfringens* consensus, were as follows: GAACnnnnGTTT, GAACnnnnGTTC, GAACnnnnnTTT, GAACnnnnnTTC. The default options were used with the limitation to 350 base pairs upstream to 35 bp downstream of a protein coding sequence. An exception was the promoter region of the putative endonuclease/exonuclease/phosphatase (MicroScope: CDR20291_2056) where we found 2 operators positioned approximately 460 upstream of the coding sequence and hence, we included the targets in the analysis. The results were subjected to manual check by extraction of gene sequences along with 1000 base pairs upstream and downstream followed by alignment and re-search of the binding sites.

### Cloning, expression and isolation of recombinant *C. difficile* LexA and RecA protein

The *C. difficile* 630 chromosomal DNA was extracted by the Genomic DNA purification kit (Thermo Scientific) according to the manufacturer`s instructions. The *lexA* and *recA* genes were amplified by PCR from the chromosomal DNA using specific primers (DinR_U 5′-GCGCGGATCCAGTGATGTTATGTATTTAGATC-3′ - DinR_D 5′-CGCACGCGTCTATTTAATAACTCTAAATAC-3′) and (RecA_U 5′-GCGCGGATCCAGTGTAGATCAAGAAAAATTAAAAG-3′ - RecA_D 5′-CGCACGCGTTTATTCTTCTACAATTTCTTTTG-3′), respectively. The PCR products were then purified and cut with *BamH*I and *Mlu*I and cloned into pET8c vector digested by the same enzyme to create plasmids pDinRCD and pRecACD for expression of proteins fusion with N-terminal His_6_ tag. Large-scale expression of proteins was performed in the *E. coli* BL21 (DE3) strain and purified from the bacterial cytoplasm by Ni-NTA affinity chromatography as described for the *E. coli* key SOS proteins [25]. PD10 desalting columns (GE Healthcare) were used for exchange of the buffer. The proteins were stored at −80°C in 20 mM NaH_2_PO4 (pH 7.4), 0.2 mM NaCl. Protein concentrations were determined using NanoDrop1000 (Thermo Scientific) and extinction coefficients at 280 nm of 7450 M^−1^ cm^−1^ for recombinant LexA and 16055 M^−1^ cm^−1^ for recombinant RecA.

### Surface plasmon resonance assays

*C. difficile* LexA-operator measurements were performed on a Biacore T100 (GE Healthcare) at 25°C as described [6]. The 3′-biotynilated 5-CGCTCGAGTAGTAAC-TEG-Bio-3′primer was immobilized on the flow cell 2 (Fc2) of the streptavidin sensor chip (GE Healthcare) in SPR buffer containing 20 mM Tris–HCl (pH 7.4), 140 mM NaCl, 0.005% surfactant P20 (GE Healthcare). To prepare double stranded DNA (dsDNA) fragments with the predicted *C. difficile* LexA operators, complementary pairs of primers presented in Additional file 3: Table S2 were dissolved in 20 mM NaH_2_PO_4_ (pH 7.4), 0.14 M NaCl and mixed in 1:1.5 (mol : mol) ratio for the longer to shorter primer, respectively. Primers were annealed in temperature gradient from 95°C to 4°C (~ 1.5 h) in PCR machine (Eppendorf). So prepared DNA fragments were approximately 22 bp duplex DNAs with 15-nucleotide overhangs complementary to the chip-immobilized primer. Approximately 44 response units of either DNA fragment were hybridised at 2 μl min^−1^ to the Fc2. The interaction of *C. difficile* LexA with the chip-immobilized DNAs was analysed by injecting repressor in SPR buffer in 20 nM concentration across the chip surface at 100 μl min^−1^ for a minute and dissociation was followed for 9 minutes. The regeneration of the surface was achieved injecting 12 s pulse of 50 mM NaOH at 100 μl min^−1^. The experiments were performed in triplicates and the representative sensorgrams are shown. Data were fitted to a 1:1 binding model to obtain the dissociation rates constants. Program MEME was used to determine LexA binding motifs [33].

SPR *C. difficile* RecA*-LexA interaction measurements were performed on a Biacore X (GE Healthcare) at 25°C as described to study the interaction among the key *E. coli* SOS proteins [25]. Experiments were performed in SPR_2 buffer (20 mM NaH_2_PO_4_ (pH 7.4), 150 mM NaCl, 2 mM MgCl_2_, 1 mM DTT, 1 mM ATP (Sigma Aldrich), 0.005% surfactant P20 (GE Healthcare). *C. diffcile* LexA repressor (2.6 μM), interacting with either the 22 bp *recA* operator DNA fragment or with the 22 bp non-specific DNA fragment derived from the *recA* operator, was passed over the sensor chip with immobilized RecA* (~2000 response units). LexA specific DNA (*recA* operator) or non-specific DNA, with 6 nucleotide changed in comparison to the specific DNA, was prepared by hybridising primers (1:1 mol to mol ratio) 5′-CAAGAGAACAAATGTTTGTAGA-3′ and 5′-TCTACAAACATTTGTTCTCTTG-3′or 5′-CAAGACCGGAAATCCTTGTAGA-3′ and 5′-TCTACAAGGATTTCCGGTCTTG-3′, respectively. The RecA*-LexA interaction was assayed at 10 μl/min for 60 s and the dissociation followed for 60 s. The sensor chip was regenerated as described [25].

### Repressor cleavage assay

Activation of either *E. coli* or *C. difficile* RecA (10 μM) nucleoprotein filament was performed on ice for 2 h as described [34]. RecA*-stimulated (~2 μM) cleavage of LexA were performed in 20 mM Tris, pH 7.4, 5 mM MgCl_2_, 1 mM ATP-γ-S (Sigma), and 1 mM DTT as described [25]. Samples were resolved on 12% SDS PAGE gels in MOPS running buffer (Invitrogen) and stained by Page blue protein stain (Thermo Scientific). The resolved bands were quantified using a G:Box (Syngene). The integrated optical densities of the LexA monomers were determined. The LexA levels throughout the time course were compared and are presented as the ratio of the density value for the sample at time indicated as 0 min relative to the density value obtained from the samples obtained later in the LexA cleavage reaction. The experiments were performed two times and representative gels are shown.

## Competing interests

The authors declare that they have no competing interests.

## Authors' contributions

BMW, NP and MB designed and performed most of the experiments, VH, NP and GA contributed to SPR experiments, NP and DZB contributed to expression and cleavage experiments; BD and MR contributed toward strain and genome selection. All authors contributed to analysis of the results and during the preparation of the manuscript.

## Supplementary Material

Additional file 1: Table S1 List of genomes used for analysis of SOS regulon and LexA variability. The names of the strains used for SOS regulon analysis are additionally bolded. Click here for file

Additional file 2: Figure S1 Comassie stained *C. difficile* (CD) LexA and RecA proteins and the LexA protein from *Escherichia coli* (EC). Proteins used in the study were more than 95% pure. Approximately 5 μg of each protein was loaded on the SDS-PAGE gel. Click here for file

Additional file 3: Table S2 Pairs of primers used to construct double stranded DNAs harbouring predicted LexA target sites. Putative LexA operators are underlined. Click here for file
