# Supplementary figures and images for: The LexA regulated genes of the Clostridium difficile
Source: BMC Microbiol. 2014 Apr 8;14:88. doi: 10.1186/1471-2180-14-88 (PMC4234289; doi:10.1186/1471-2180-14-88)

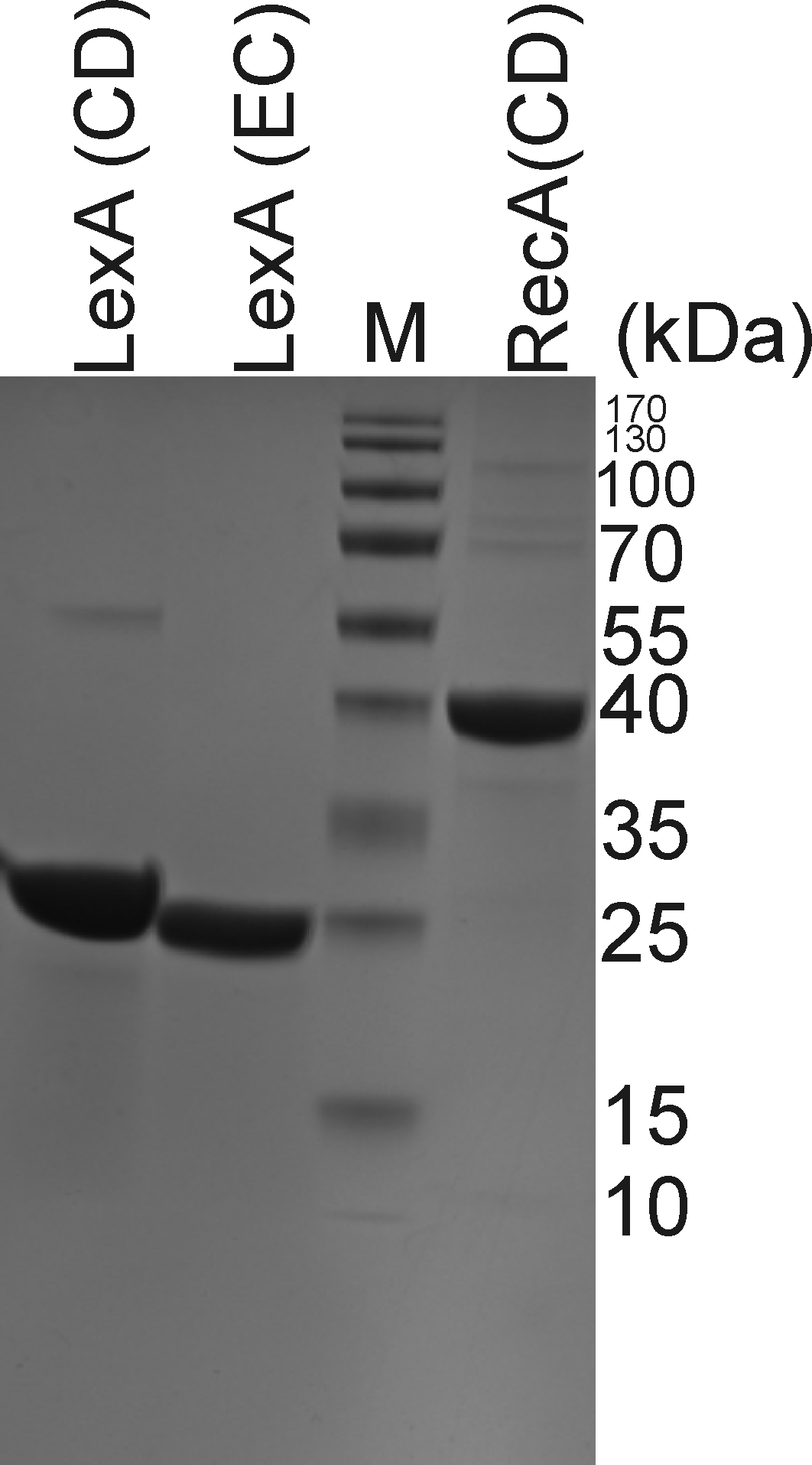

Supplement: Additional file 2: Figure S1 — Comassie stained C. difficile (CD) LexA and RecA proteins and the LexA protein from Escherichia coli (EC). Proteins used in the study were more than 95% pure. Approximately 5 μg of each protein was loaded on the SDS-PAGE gel. [file 1471-2180-14-88-S2.tiff]
